# Surgical Management of Pilonidal Sinus: A Comparative Study of Excision, Limberg Flap, and Keystone Flap Reconstruction

**DOI:** 10.7759/cureus.114137

**Published:** 2026-08-07

**Authors:** Monya Punyo, Moorat Singh Yadav, Deepak Krishna, Ajeet P Maurya, Surya Jain, Jaya Jain

**Affiliations:** 1 General Surgery, All India Institute of Medical Sciences Bhopal, Bhopal, IND; 2 Burns and Plastic Surgery, All India Institute of Medical Sciences Bhopal, Bhopal, IND

**Keywords:** flap reconstruction, keystone flap, limberg flap, off-midline closure, pilondal sinus, pilonidal sinus surgery, quality of life (qol), ­wound healing

## Abstract

Background

Pilonidal sinus is a chronic inflammatory condition predominantly affecting young adult men, with significant recurrence and morbidity. Multiple surgical techniques exist, but no single method has been universally accepted as optimal, particularly for managing recurrence, complications, and quality-of-life (QoL) outcomes.

Methods

This prospective comparative observational study enrolled 46 patients with pilonidal sinus disease who underwent either simple excision, Limberg flap, or Keystone flap reconstruction at a tertiary care center between February 2024 and July 2025. Patients were followed at one, three, and six weeks postoperatively, and outcomes assessed included operative time, hospital stay, healing duration, complications classified by Clavien-Dindo, recurrence rates, scar quality using the Vancouver Scar Scale, patient satisfaction on a Likert scale, and QoL measured by WHO‑QoL‑BREF.

Results

The cohort showed male predominance (89.1%) with a mean age of 25.24 ± 6.16 years. Flap procedures were used for more extensive disease and had longer operative time and hospital stay (p < 0.001). Healing duration was comparable, though simple excision showed longer median healing time (p = 0.031). Return to work was earlier with simple excision, but the difference was not statistically significant (p = 0.057). Complications occurred in 43.5% of patients, most commonly seroma (28.3%), with higher rates in flap groups, while recurrence (13.3%) was seen only in the simple excision group (p < 0.001). Flap procedures demonstrated superior scar outcomes (p = 0.014) and higher patient satisfaction (p = 0.002). QoL assessment showed significantly better physical (p < 0.001) and psychological (p = 0.002) scores in flap groups, with no difference in social and environmental domains.

Conclusion

While flap procedures are associated with longer operative time and hospital stay, they provide superior long-term outcomes in terms of recurrence, cosmesis, patient satisfaction, and QoL. Off-midline flap techniques, particularly Limberg and keystone flaps, are preferable for complex or recurrent pilonidal sinus disease.

## Introduction

Pilonidal sinus disease is a chronic inflammatory condition most commonly affecting the sacrococcygeal region, with an annual incidence of approximately 26 per 100,000 individuals. It predominantly occurs in young adults aged 15-30 years, with a male-to-female ratio ranging from 2.2:1 to 4:1 [1]. Clinically, it presents as one or more midline pits communicating with a fibrous tract containing hair and granulation tissue [2]. The spectrum of presentation varies from asymptomatic cases to acute abscess formation, recurrent pain, and itching. Rarely, malignant transformation of the sinus tract has been reported in long-standing disease [3].

Risk factors include male gender, sedentary lifestyle, family history, coarse hair, deep natal cleft, poor hygiene, and skin sensitivity [4]. Pilonidal sinus is now considered an acquired condition, resulting from hair penetration into the natal cleft under mechanical forces, leading to chronic inflammation and sinus formation [5].

The disease imposes a significant socioeconomic burden in young working-age populations due to recurrence rates approaching 50%, prolonged morbidity, and occasional malignant transformation [6,7]. While non-surgical options such as hair removal, laser epilation, and fibrin glue have been explored, definitive management remains surgical [8-10]. The principle of surgery is complete eradication of the sinus tract, achieved through techniques such as simple excision, primary closure, and flap reconstructions, including Karydakis, Limberg, Bascom, and keystone flaps.

Simple excision offers lower recurrence rates (5-15%) and fewer wound complications compared to primary closure but requires prolonged dressing care and may leave a large scar [1,11]. The Limberg flap provides robust vascularity, low recurrence rates (~2-5%), and favorable cosmetic outcomes, but demands greater surgical expertise and carries the risk of flap necrosis [12,13]. The keystone flap, in contrast, allows shorter operative and healing times with a lower recurrence rate, though immediate postoperative complications and flap necrosis are slightly higher [14].

The ideal surgery for this disease should have minimal excision, a low recurrence rate, a shorter hospital stay, minimal loss of labor, and minimal scar [15]. Although various surgical techniques have emerged over time, there is no consensus on a single ideal method. Thus, a comparative study was planned to compare the demographic, intraoperative, and postoperative outcomes in patients with pilonidal sinus undergoing simple excision, keystone flap, and Limberg flap.

## Materials and methods

This was a prospective, comparative, observational study conducted at a tertiary care institute in India from February 2024 to July 2025. The aim of the study was to compare outcomes in patients with Pilonidal sinus undergoing simple excision, the Keystone flap, or the Limberg flap.

Forty-six patients diagnosed with pilonidal sinus were included in the study and underwent surgery with either of the above-mentioned surgical options. The study included patients diagnosed with pilonidal sinus who gave informed consent, were above 18 years of age, and were undergoing surgery for pilonidal sinus using any one of the above-mentioned three techniques. Recurrent pilonidal sinus disease patients were also included. Patients with acute abscesses who were unwilling to undergo the procedure were excluded.

The study commenced only after obtaining approval from the Institutional Ethics Committee. Written informed consent was obtained from every participant prior to enrolment.

Patients with pilonidal sinus disease were admitted to the general surgery department and allocated to three study groups by the covariate adaptive method. Their clinical and demographic details, along with relevant radiology (USG/MRI) and blood investigations, were recorded, and a pre-operative work-up was performed. They were followed up at the first, third, and sixth weeks postoperatively. Study outcomes were measured in all three groups, and results were compared. Primary outcomes were operation time (in minutes), postoperative healing time, postoperative complications (using the Clavien-Dindo classification) [16], and recurrence of disease, which were compared across the mentioned surgical techniques. Aesthetic outcomes (using a Likert scale) [17], postoperative quality of life (using the WHOQOL-BREF questionnaire) [18], and scar (using the Vancouver Scar Scale) [19] were among the secondary outcomes assessed.

The chi-square test was used to compare groups for categorical data. In case the expected frequency in the contingency tables was found to be <5 for >25% of the cells, Fisher’s exact test was used instead. Linear correlation between two continuous variables was explored using Pearson’s correlation (if the data are normally distributed) and Spearman’s correlation (for non-normally distributed data). Continuous variables were compared across the three groups using one-way analysis of variance (ANOVA) for normally distributed data and the Kruskal-Wallis test for non-normally distributed data, with normality assessed using the Shapiro-Wilk test. Statistical significance was kept at p < 0.05.

Surgical methods

Primary Excision

**Figure 1:**
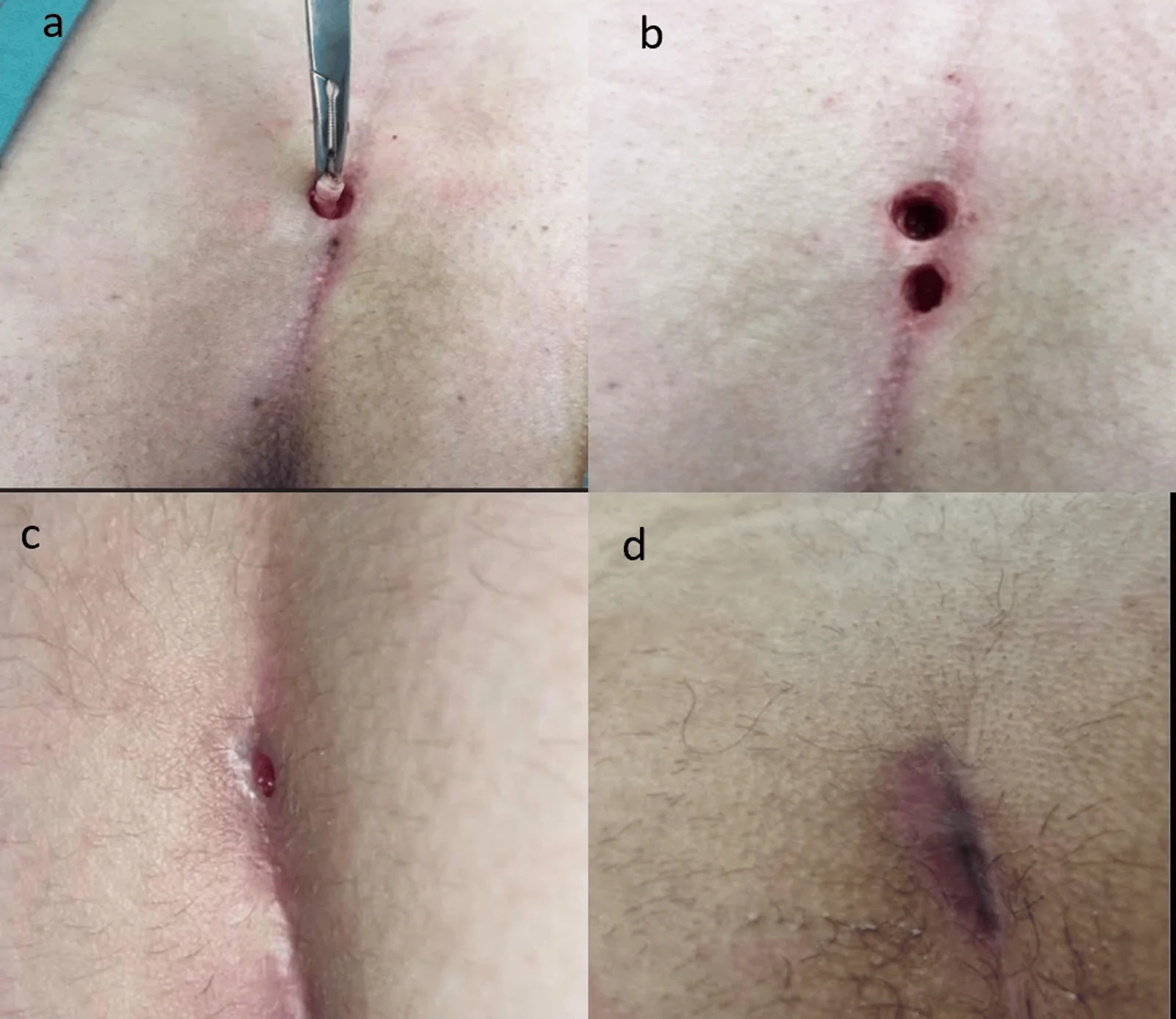
Simple (primary) excision of pilonidal sinus Intraoperative and follow-up photographs of simple (primary) excision of the pilonidal sinus: (a) complete excision of the sinus together with a margin of healthy tissue; (b) wound left open to heal by secondary intention; (c) follow-up at four weeks; (d) follow-up at three months

Primary excision involved the complete removal of the pilonidal sinus and its tracts, with the wound left open to heal by secondary intention through granulation and epithelialization [11]. Wide elliptical excision of the sinus and surrounding tissue down to the sacrococcygeal fascia was performed, and the wound was left open to heal by granulation, contraction, and epithelialization with regular dressing (Figures 1a-1d).

Keystone Flap

**Figure 2:**
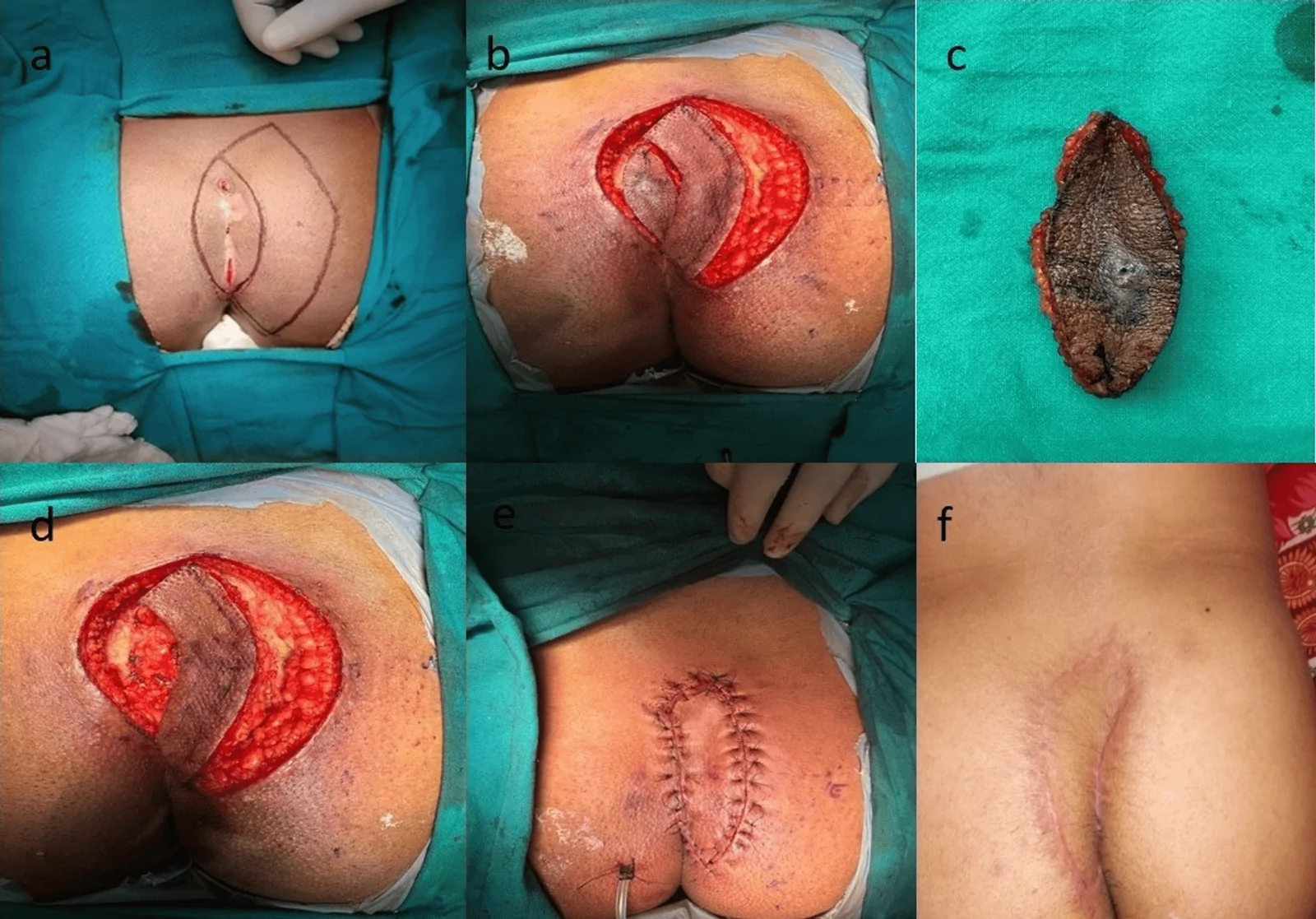
Keystone perforator-island flap reconstruction Intraoperative and follow-up photographs illustrating the sequential steps of keystone perforator-island flap reconstruction for sacrococcygeal pilonidal sinus disease: (a) flap marking; (b) excision of the pilonidal sinus; (c) excised tissue specimen; (d) flap mobilization; (e) flap inset with drain placement; (f) follow-up at three months.

It is a perforator-based local advancement flap (a subtype of perforator island flaps), remaining largely contiguous with surrounding tissues. The flap was marked by drawing tangent lines from each apex and connecting them with an arc. After complete excision of diseased tissue to healthy fascia, the flap was elevated, advanced into the defect without tension, preserving perforators, and closure was performed over a drain using interrupted sutures, starting at the point of greatest tension (Figure 2a-2f) [14].

Limberg Flap

**Figure 3:**
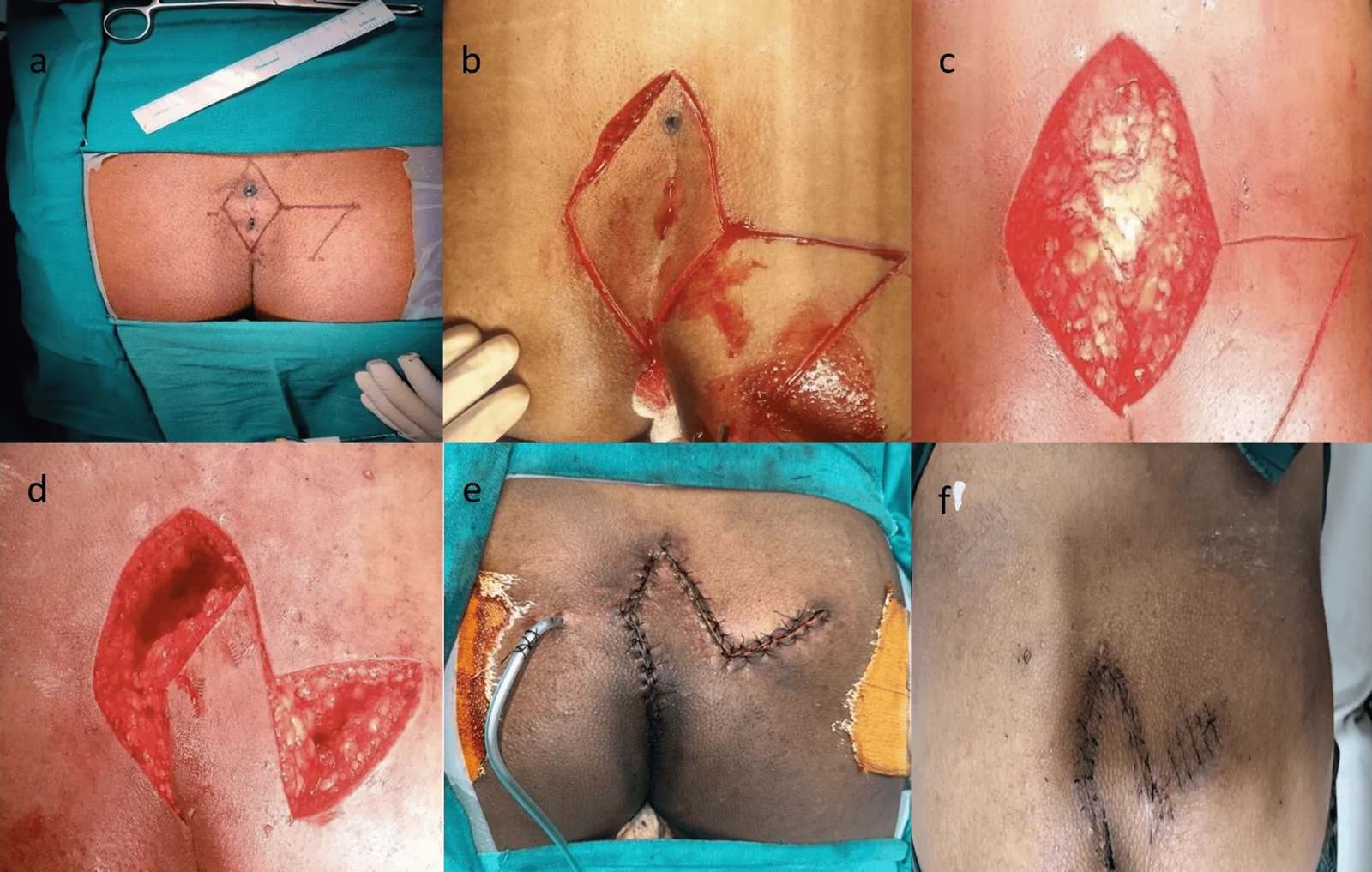
Limberg (rhomboid transposition) flap reconstruction Intraoperative and follow-up photographs illustrating the sequential steps of Limberg (rhomboid transposition) flap reconstruction for sacrococcygeal pilonidal sinus disease: (a) flap marking; (b) excision of the pilonidal sinus; (c) post-excision defect; (d) flap mobilization; (e) flap inset with drain placement; (f) postoperative day 3.

The Limberg flap is a rhomboid transposition flap used to cover post-excisional defects, providing off-midline closure that reduces tension, moisture, and the risk of recurrence. The defect was excised in a rhomboid shape, with its long axis in the midline and its short axis transverse, ensuring complete removal of sinus tracts down to the presacral fascia and well-vascularized edges. A fasciocutaneous flap was then elevated, preserving perforators; transposed without tension to cover the defect; and secured in layers over a drain to prevent seroma or hematoma (Figures 3a-3f) [12].

## Results

A total of 46 participants were included: 16 underwent the Limberg flap, 15 underwent the keystone flap, and 15 underwent simple excision. The cohort showed marked male predominance (89.1%) with a mean age of 25.24 ± 6.16 years, most commonly in the 20-29 age group (56.5%). Students comprised 50% of the population, and the majority (93.5%) had no comorbidities (Table 1).

**Table 1:** Comparison of baseline demographic, clinical, and postoperative characteristics among the three surgical groups This table summarizes the baseline demographic, clinical, and postoperative characteristics of the 46 study participants across the Limberg flap, Keystone flap, and simple excision groups. Overall, the groups were comparable in most baseline variables, with significant differences only in educational status (p = 0.022), discharge on examination (p = 0.014), and the overall complication rate (p = 0.016). Data are presented as mean ± SD (continuous variable) or n (%) (categorical variables). Continuous variables were compared using the Kruskal–Wallis test and categorical variables using the chi-square or Fisher's exact test; the χ² column reports the Pearson chi-square statistic for each comparison. A dash (–) denotes not applicable. SD: standard deviation; χ²: chi-square statistic; T2DM: type 2 diabetes mellitus; p: probability value. ^1^Kruskal–Wallis test; ²Fisher's exact test; ³chi-square test. SD: standard deviation; T2DM: type 2 diabetes mellitus; p, probability value.

Parameters	Limberg (n = 16)	Keystone (n = 15)	Simple excision (n = 15)	χ²	p-value
Age, years (mean ± SD) ¹	27.94 ± 6.95	22.53 ± 4.84	25.07 ± 5.52	–	0.066
Age group ²	–	–	–	4.978	0.612
<20 years	3 (18.8%)	4 (26.7%)	2 (13.3%)	–	–
20–29 years	7 (43.8%)	8 (53.3%)	11 (73.3%)	–	–
30–39 years	5 (31.2%)	3 (20.0%)	2 (13.3%)	–	–
40–49 years	1 (6.2%)	0 (0.0%)	0 (0.0%)	–	–
Gender ³	–	–	–	0.411	1
Male	14 (87.5%)	14 (93.3%)	13 (86.7%)	–	–
Female	2 (12.5%)	1 (6.7%)	2 (13.3%)	–	–
Occupation ²	–	–	–	19.687	0.819
Education ²	–	–	–	19.11	0.022
Student	8 (50.0%)	9 (60.0%)	6 (40.0%)	–	–
Primary	0 (0.0%)	0 (0.0%)	4 (26.7%)	–	–
High school	4 (25.0%)	0 (0.0%)	0 (0.0%)	–	–
Diploma	0 (0.0%)	0 (0.0%)	1 (6.7%)	–	–
Graduate	4 (25.0%)	6 (40.0%)	4 (26.7%)	–	–
Comorbidities ³	–	–	–	5.946	1
None	14 (87.5%)	15 (100.0%)	14 (93.3%)	–	–
Hypertension	1 (6.2%)	0 (0.0%)	0 (0.0%)	–	–
Obesity	1 (6.2%)	0 (0.0%)	0 (0.0%)	–	–
T2DM	0 (0.0%)	0 (0.0%)	1 (6.7%)	–	–
Site of pits in natal cleft, n (%) ³	16 (100.0%)	15 (100.0%)	15 (100.0%)	0	1
Tenderness ³	–	–	–	1.917	1
No	15 (93.8%)	15 (100.0%)	15 (100.0%)	–	–
Minimal	1 (6.2%)	0 (0.0%)	0 (0.0%)	–	–
Discharge ²	–	–	–	8.493	0.014
No	15 (93.8%)	11 (73.3%)	7 (46.7%)	–	–
Minimal	1 (6.2%)	4 (26.7%)	8 (53.3%)	–	–
Any complication, n (%) ²	9 (56.2%)	9 (60.0%)	2 (13.3%)	8.275	0.016
Need for wound therapy, n (%) ³	2 (12.5%)	1 (6.7%)	1 (6.7%)	0.447	1
Failure of healing at 12 weeks, n (%) ³	1 (6.2%)	0 (0.0%)	0 (0.0%)	1.917	1
Recurrence, n (%) ³	0 (0.0%)	0 (0.0%)	2 (13.3%)	4.321	0.203

97.8% of participants had no tenderness, and around 28.3% had minimal discharge on examination (Table 1). Limberg flap required the longest duration, and simple excision the shortest (p < 0.001) (Table 2).

**Table 2:** Comparison of operative duration among the Limberg, Keystone, and simple excision groups This table compares the operative duration (in minutes) among the three procedure groups. Operative duration was significantly longer for the flap procedures, being longest for the Limberg flap and shortest for simple excision (p < 0.001). Data are presented as mean (SD), median (IQR), and minimum–maximum. SD: standard deviation; IQR: interquartile range; χ²: chi-square statistic of the Kruskal–Wallis test. Statistical significance was set at p < 0.05. p, probability value.

Statistic	Limberg (n = 16)	Keystone (n = 15)	Simple excision (n = 15)	χ²	p-value
Mean (SD)	135.75 (16.67)	100.40 (18.35)	33.33 (10.80)	37.399	<0.001
Median (IQR)	135 (120-150)	90 (90-120)	30 (30-37.5)	–	–
Min - Max	108 - 168	60 - 120	20 - 60	–	–

Excised defect areas were significantly larger in flap procedures (Limberg and Keystone) compared to simple excision, reflecting their use in patients with more extensive disease (p < 0.001) (Table 3).

**Table 3:** Comparison of the excised defect area among the three surgical groups This table compares the excised defect area (cm²) among the three procedure groups. The excised defect was significantly larger in the two flap groups than in the simple excision group (p < 0.001). Data are presented as mean (SD), median (IQR), and minimum–maximum. SD: standard deviation; IQR: interquartile range; χ²: chi-square statistic of the Kruskal–Wallis test. Statistical significance was set at p < 0.05. p, probability value.

Statistic	Limberg (n = 16)	Keystone (n = 15)	Simple excision (n = 15)	χ²	p-value
Mean (SD)	23.62 (14.23)	22.48 (12.49)	2.81 (1.77)	29.777	<0.001
Median (IQR)	18.12 (16-26.31)	18 (16.5-26.25)	2.25 (1.44-4)	–	–
Min - Max	9 - 64	8.75 - 60	1 - 6.25	–	–

**Figure 4:**
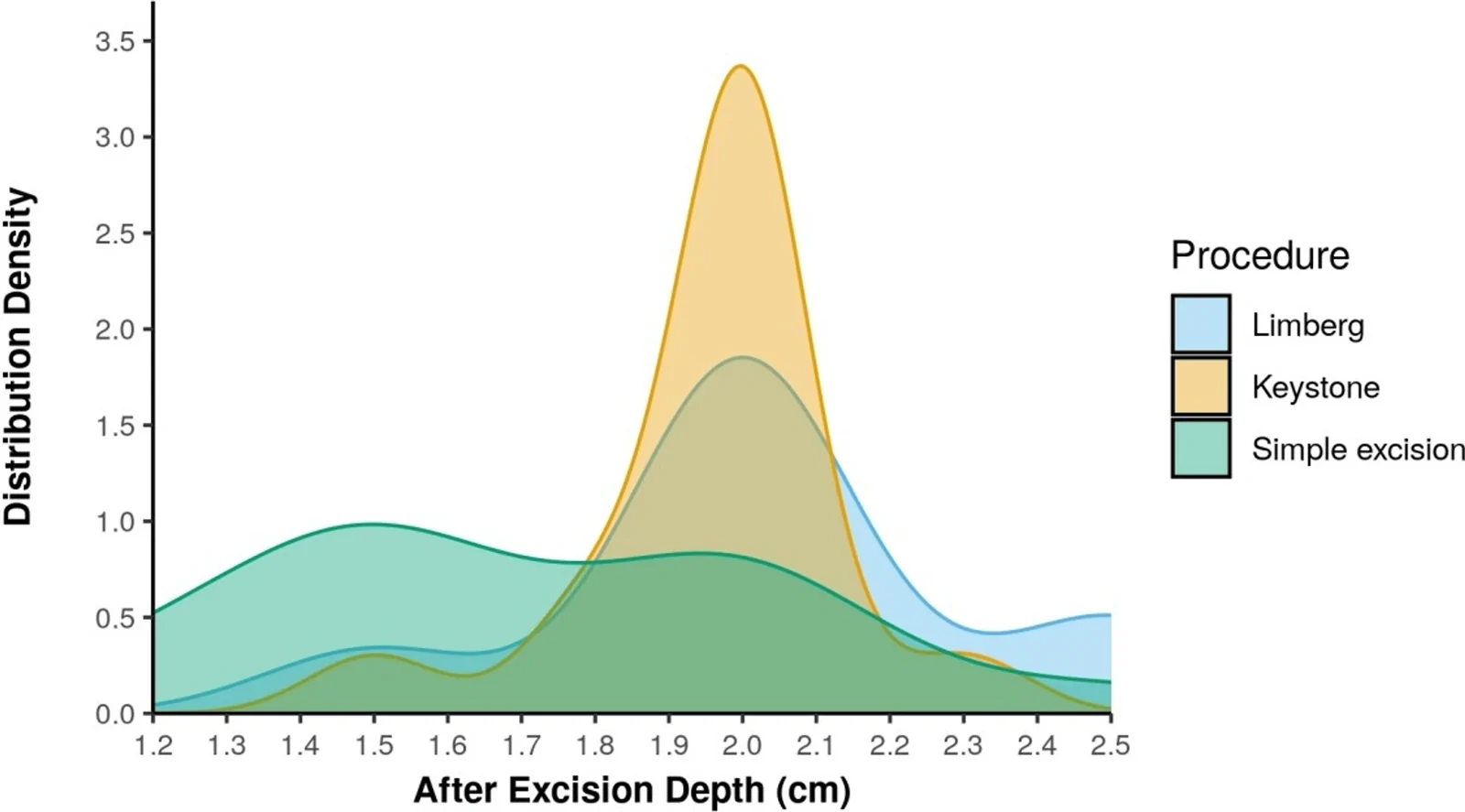
Comparison of the depth of the excised defect among the three surgical groups Kernel density plot comparing the depth of the excised defect (cm) across the three procedure groups, showing the greatest excision depth in the Limberg flap group. Kruskal–Wallis test: χ² = 8.883, p = 0.012. χ², chi-square test statistic; p, probability value.

A statistically significant difference was noted between the groups (p = 0.012) for defect depth (cm), with the Limberg group having the greatest mean excision depth. Importantly, for flap procedures (Limberg and keystone), the excision depth was influenced more by the anatomical depth of the buttock cleft rather than by the flap type itself (Figure 4).

**Figure 5:**
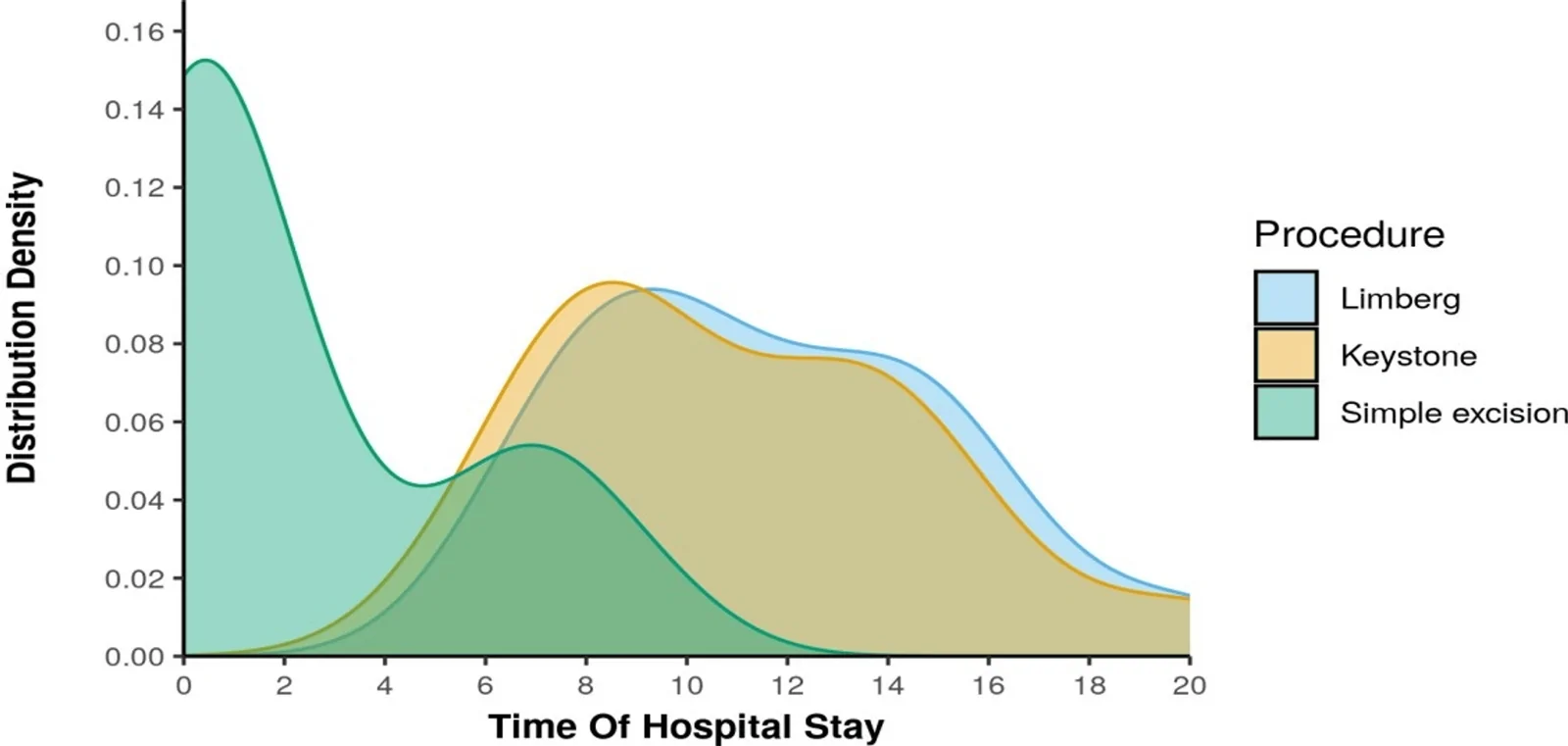
Comparison of postoperative hospital stay among the three surgical groups Kernel density plot comparing the duration of postoperative hospital stay (in days) across the three procedure groups, with the longest stay after the Limberg flap and the shortest after simple excision. Kruskal–Wallis test: χ² = 26.115, p < 0.001. χ², chi-square test statistic; p, probability value.

A significant difference in hospital stay was observed between the groups (p < 0.001), with the Limberg group having the longest duration, reflecting the more extensive nature of the procedure compared to simple excision, which permitted earlier discharge (Figure 5).

**Figure 6:**
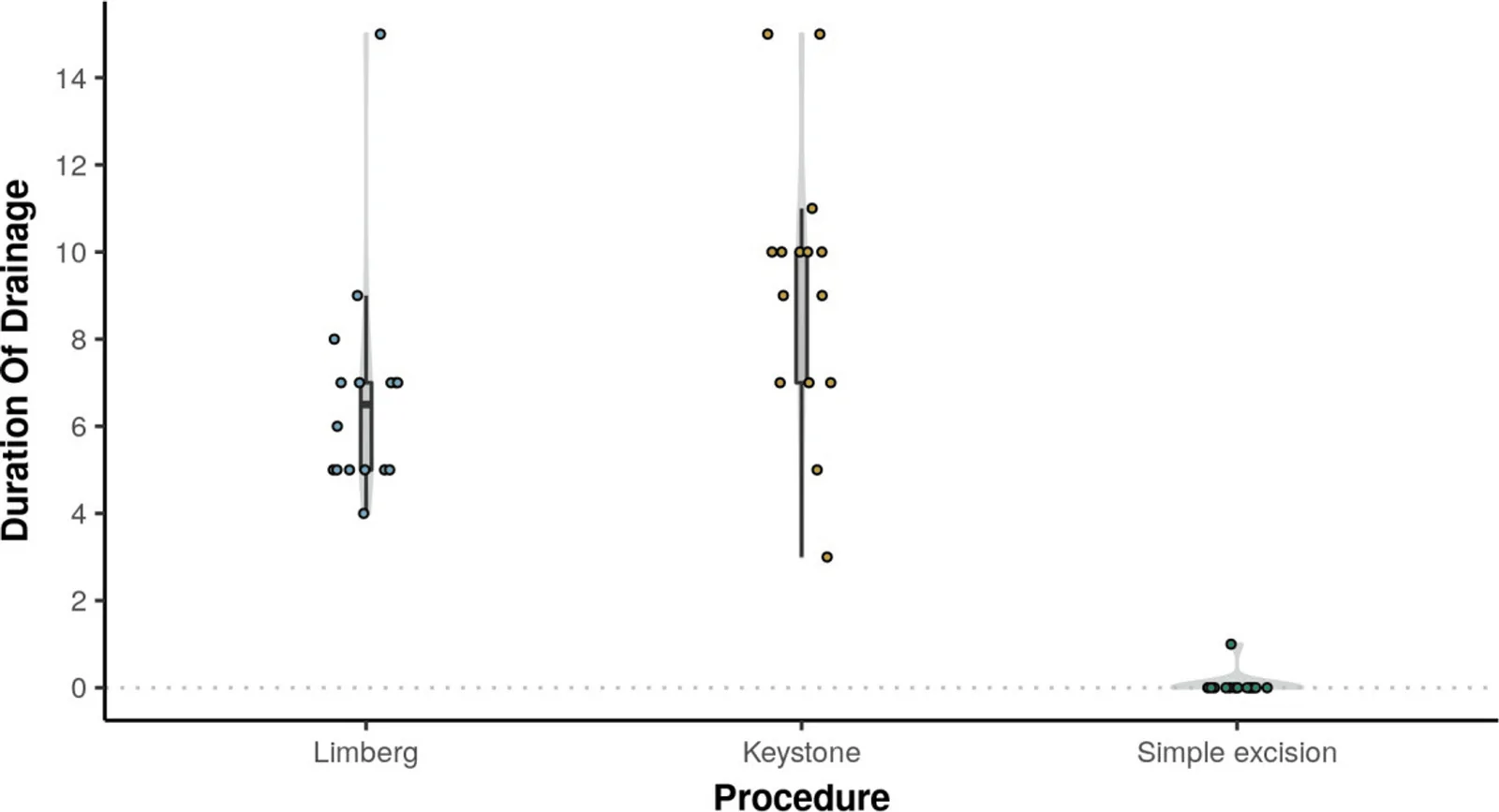
Comparison of the duration of postoperative drainage among the three surgical groups Dot plot with error bars (mean ± SD) comparing the duration of postoperative wound drainage (in days) across the three procedure groups, which was largely confined to the flap procedures. Kruskal–Wallis test: χ² = 34.065, p < 0.001. SD, standard deviation; χ², chi-square test statistic; p, probability value.

The mean drainage duration was 6.69 ± 2.60 days in the Limberg group, 9.20 ± 3.21 days in the keystone group, and negligible in the simple excision group, with a statistically significant difference observed (p < 0.001) (Figure 6).

**Figure 7:**
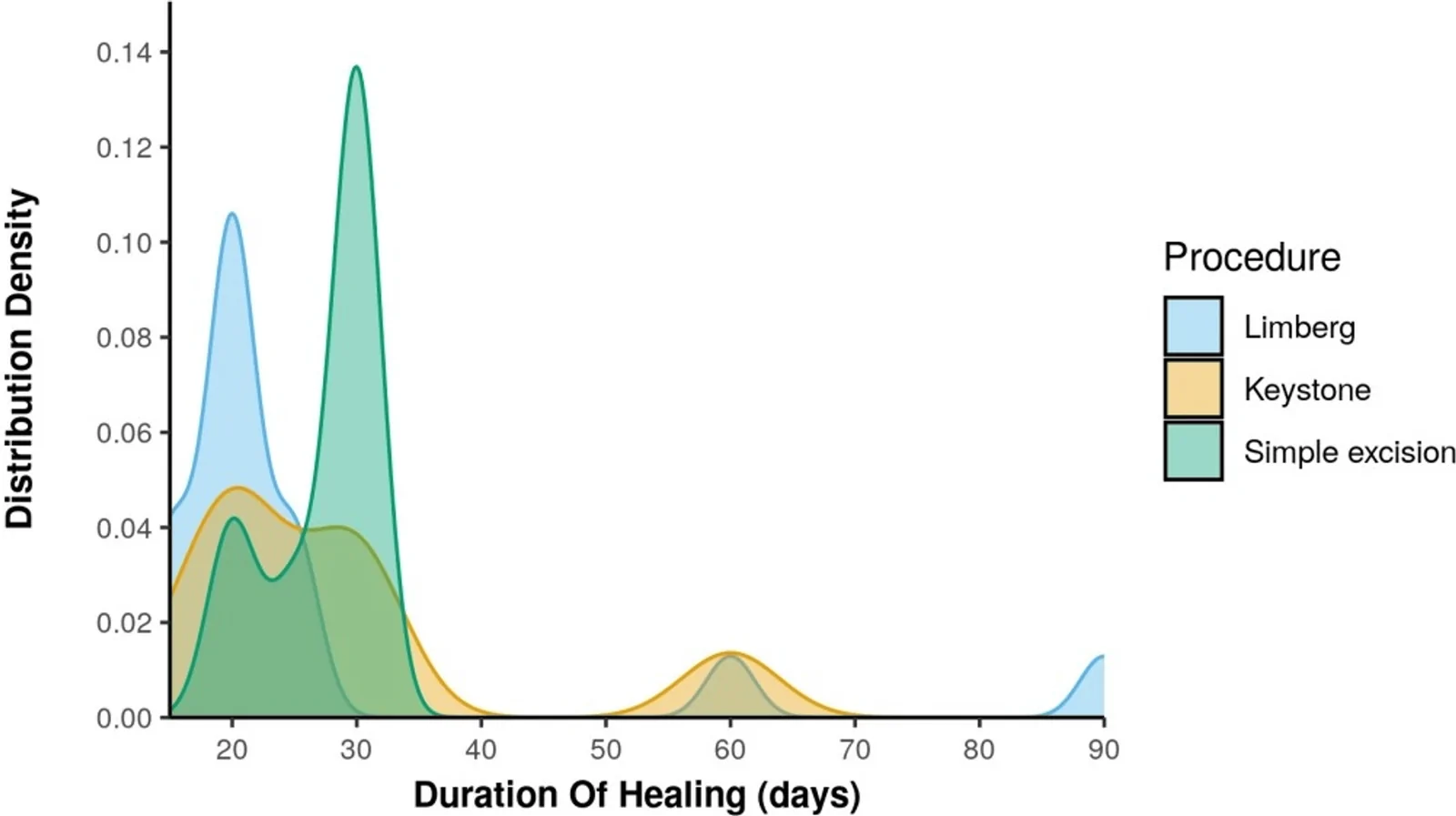
Comparison of the duration of wound healing among the three surgical groups Kernel density plot comparing the duration of wound healing (in days) across the three procedure groups, with the longest median healing time in the simple excision group. Kruskal–Wallis test: χ² = 6.943, p = 0.031. χ², chi-square test statistic; p, probability value.

Mean healing duration was similar across groups, but the simple excision group had the longest median healing time (p = 0.031) (Figure 7).

Return to work was earlier in the simple excision group (23.00 ± 5.61 days) than in the Limberg (32.50 ± 18.71 days) and keystone (31.67 ± 12.49 days) groups; however, the difference was not statistically significant (p = 0.057) (Table 4).

**Table 4:** Comparison of the time to return to work among the three surgical groups This table compares the time taken to return to work (in days) among the three procedure groups. Return to work was earliest after simple excision, although the difference between groups was not statistically significant (p = 0.057). Data are presented as mean (SD), median (IQR), and minimum–maximum. SD: standard deviation; IQR: interquartile range; χ²: chi-square statistic of the Kruskal–Wallis test. Statistical significance was set at p < 0.05. p, probability value.

Statistic	Limberg (n = 16)	Keystone (n = 15)	Simple excision (n = 15)	χ²	p-value
Mean (SD)	32.50 (18.71)	31.67 (12.49)	23.00 (5.61)	5.742	0.057
Median (IQR)	30 (20-30)	30 (25-30)	20 (20-30)	–	–
Min - Max	20 - 90	20 - 60	15 - 30	–	–

**Figure 8:**
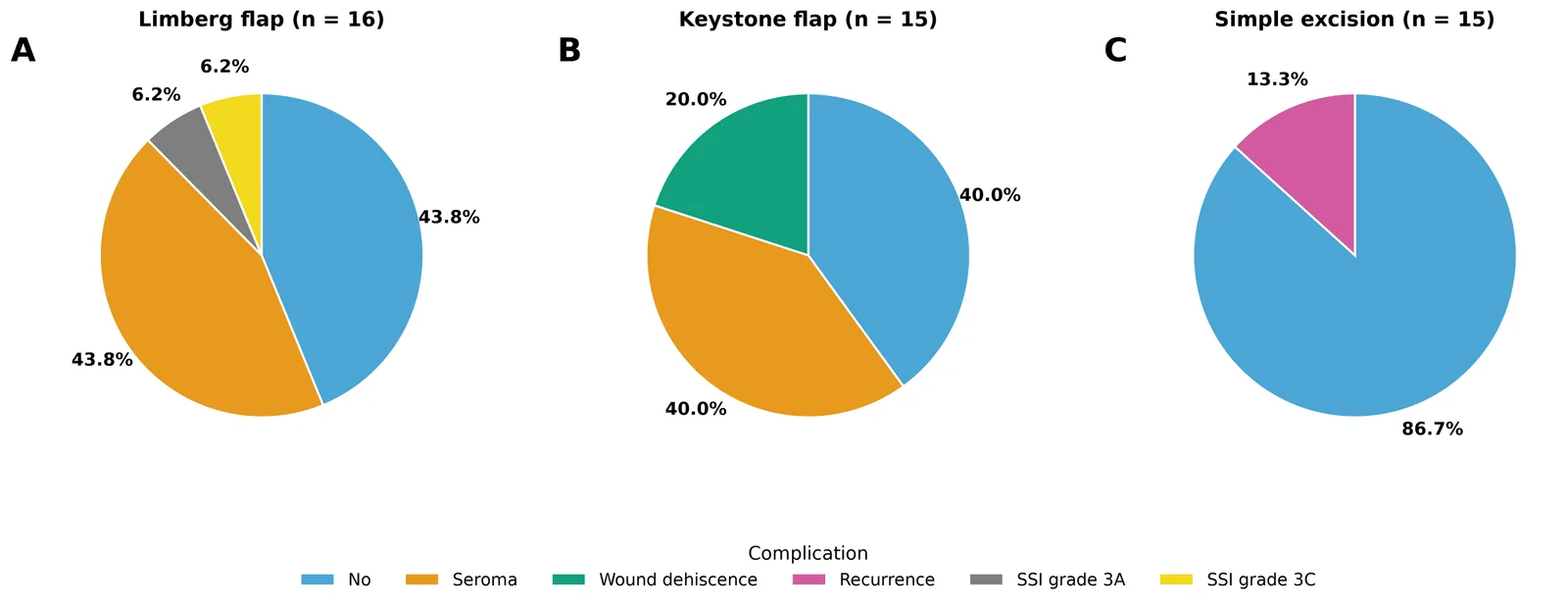
Distribution of postoperative complications within each surgical group Pie charts depicting the distribution of postoperative complications within each procedure group, showing more procedure-related complications after the flap procedures and a higher recurrence rate after simple excision. Fisher's exact test: χ² = 24.010, p < 0.001. χ², chi-square test statistic; p, probability value.

Overall, over half of the patients (56.5%) had no complications, while seroma (28.3%) was most common, followed by wound dehiscence (6.5%) and recurrence (4.3%), with a significant association between groups (p < 0.001). Flap procedures showed more procedure-related complications (seroma, dehiscence), whereas simple excision had fewer immediate complications but a higher recurrence rate (Figure 8).

Scar outcomes, measured by the Vancouver Scar Scale, differed significantly between groups (p = 0.014). The simple excision group had the highest scores, indicating less favorable scarring, whereas the Limberg and keystone groups demonstrated lower scores, reflecting better cosmetic results (Table 5).

**Table 5:** Comparison of postoperative scar quality (Vancouver Scar Scale) among the three surgical groups This table compares postoperative scar quality, assessed using the Vancouver Scar Scale, among the three procedure groups. Scar quality was significantly better (lower scores) after the flap procedures than after simple excision (p = 0.014). Data are presented as mean (SD), median (IQR), and minimum–maximum. SD: standard deviation; IQR: interquartile range; χ²: chi-square statistic of the Kruskal–Wallis test. Statistical significance was set at p < 0.05. p, probability value. Higher Vancouver Scar Scale scores indicate poorer scar quality.

Statistic	Limberg (n = 16)	Keystone (n = 15)	Simple excision (n = 15)	χ²	p-value
Mean (SD)	5.44 (1.50)	5.53 (1.36)	7.07 (1.75)	8.547	0.014
Median (IQR)	5 (5-6)	5 (5-6.5)	7 (6-8.5)	–	–
Min - Max	3 - 10	3 - 8	4 -10	–	–

A significant difference in Likert scores was observed (p = 0.002), with the Limberg group reporting the highest patient satisfaction compared to keystone and simple excision (Table 6).

**Table 6:** Comparison of patient satisfaction (Likert scale) among the three surgical groups This table compares patient-reported satisfaction, measured on the Likert scale, among the three procedure groups. Patient satisfaction was significantly higher after the flap procedures, particularly the Limberg flap (p = 0.002). Data are presented as mean (SD), median (IQR), and minimum–maximum. SD: standard deviation; IQR: interquartile range; χ²: chi-square statistic of the Kruskal–Wallis test. Statistical significance was set at p < 0.05. p, probability value. Higher Likert scores indicate greater patient satisfaction.

Statistic	Limberg (n = 16)	Keystone (n = 15)	Simple excision (n = 15)	χ²	p-value
Mean (SD)	8.54 (0.54)	8.24 (0.66)	7.62 (0.67)	12.174	0.002
Median (IQR)	8.4 (8.2-9.03)	8.1 (7.9-8.65)	7.7 (7.25-8)	–	–
Min - Max	7.6 - 9.5	6.9 - 9.4	6.4 - 8.7	–	–

**Figure 9:**
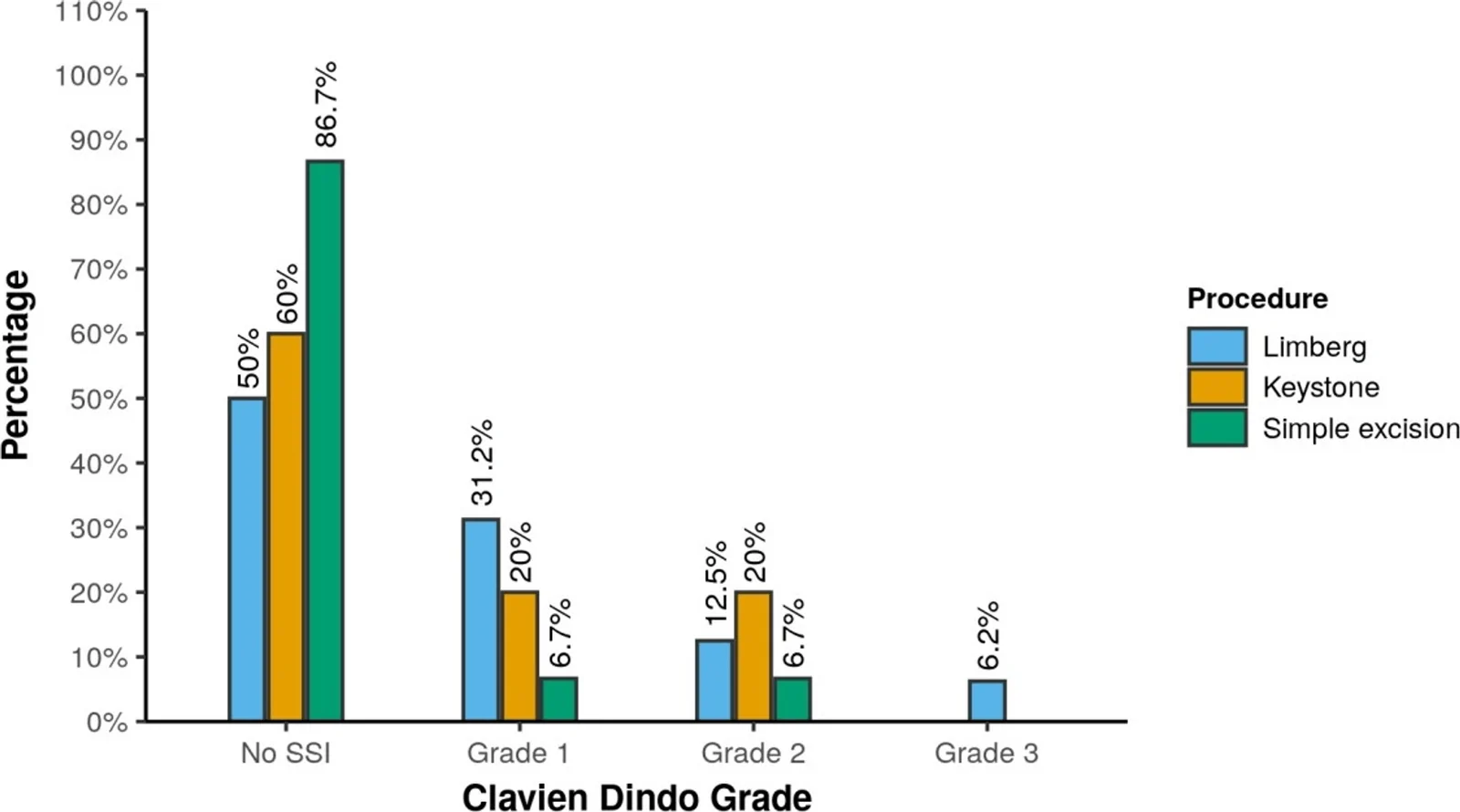
Comparison of the Clavien–Dindo grade of postoperative complications among the three surgical groups Clustered bar chart comparing the Clavien–Dindo grade of postoperative complications across the three procedure groups, with no significant difference between the techniques. Fisher's exact test: χ² = 6.985, p = 0.309. χ², chi-square test statistic; p, probability value.

No significant difference was observed in Clavien-Dindo grades (p = 0.309); Limberg and keystone flaps had similar SSI rates, while simple excision had fewer SSIs but higher recurrence rates (Figure 9).

The WHOQOL-BREF: Physical quality of life (QoL) scores differed significantly between groups (p < 0.001), with the highest values in the keystone group; both keystone and Limberg flaps showed better physical QoL than simple excision (Table 7).

**Table 7:** Comparison of physical-domain quality of life (WHO-QoL-BREF) among the three surgical groups This table compares the physical-domain scores of the WHO-QoL-BREF questionnaire among the three procedure groups. Physical-domain quality of life was significantly higher in the flap groups, especially the Keystone flap, than after simple excision (p < 0.001). Data are presented as mean (SD), median (IQR), and minimum–maximum. SD: standard deviation; IQR: interquartile range; F: one-way ANOVA F statistic; WHO-QoL-BREF: World Health Organization Quality of Life–BREF questionnaire. Statistical significance was set at p < 0.05. p, probability value.

Statistic	Limberg (n = 16)	Keystone (n = 15)	Simple excision (n = 15)	F	p-value
Mean (SD)	72.67 (7.59)	76.39 (7.76)	65.10 (5.76)	9.847	<0.001
Median (IQR)	71.3 (67.1-76)	75.2 (72.7-80.95)	66 (60.25-69.4)	–	–
Min – Max	63.2 – 89.7	61.4 – 88.6	54.6 – 73.8	–	–

Psychological QoL differed significantly among groups (p = 0.002), with the highest scores in the Limberg group; flap procedures showed better psychological outcomes than simple excision (Table 8).

**Table 8:** Comparison of psychological-domain quality of life (WHO-QoL-BREF) among the three surgical groups This table compares the psychological-domain scores of the WHO-QoL-BREF questionnaire among the three procedure groups. Psychological-domain quality of life was significantly higher in the flap groups, particularly the Limberg flap (p = 0.002). Data are presented as mean (SD), median (IQR), and minimum–maximum. SD: standard deviation; IQR: interquartile range; F: one-way ANOVA F statistic; WHO-QoL-BREF: World Health Organization Quality of Life–BREF questionnaire. Statistical significance was set at p < 0.05. p, probability value.

Statistic	Limberg (n = 16)	Keystone (n = 15)	Simple excision (n = 15)	F	p-value
Mean (SD)	78.47 (7.01)	78.40 (5.09)	70.97 (6.05)	7.533	0.002
Median (IQR)	78.65 (76.35-82.8)	80 (75.3-82.8)	69.9 (67.75-74.45)	–	–
Min – Max	60.2 – 87.4	67.1 – 84.6	57.5 – 81.7	–	–

Social QoL scores were comparable across groups, with no significant difference observed (p = 0.146), indicating minimal impact of surgical type on social functioning (Table 9).

**Table 9:** Comparison of social-domain quality of life (WHO-QoL-BREF) among the three surgical groups This table compares the social-domain scores of the WHO-QoL-BREF questionnaire among the three procedure groups. Social-domain quality of life was comparable across the three groups, with no significant difference (p = 0.146). Data are presented as mean (SD), median (IQR), and minimum–maximum. SD: standard deviation; IQR: interquartile range; F: one-way ANOVA F statistic; WHO-QoL-BREF: World Health Organization Quality of Life–BREF questionnaire. Statistical significance was set at p < 0.05. p, probability value.

Statistic	Limberg (n = 16)	Keystone (n = 15)	Simple excision (n = 15)	F	p-value
Mean (SD)	75.34 (7.50)	74.12 (4.73)	71.09 (5.41)	2.016	0.146
Median (IQR)	74.9 (71.52-79.15)	73.9 (72.5-76.4)	69.9 (67-74.15)	–	–
Min - Max	62.1 - 88.8	66.3 - 86	63.2 - 83	–	–

Environmental QoL scores were similar across groups, with no significant difference (p = 0.449), suggesting minimal impact of surgical technique on this domain (Table 10).

**Table 10:** Comparison of environmental-domain quality of life (WHO-QoL-BREF) among the three surgical groups This table compares the environmental-domain scores of the WHO-QoL-BREF questionnaire among the three procedure groups. Environmental-domain quality of life was comparable across the three groups, with no significant difference (p = 0.449). Data are presented as mean (SD), median (IQR), and minimum–maximum. SD: standard deviation; IQR: interquartile range; F: one-way ANOVA F statistic; WHO-QoL-BREF: World Health Organization Quality of Life–BREF questionnaire. Statistical significance was set at p < 0.05. p, probability value.

Statistic	Limberg (n = 16)	Keystone (n = 15)	Simple excision (n = 15)	F	p-value
Mean (SD)	72.69 (5.32)	70.61 (4.54)	72.63 (5.41)	0.815	0.449
Median (IQR)	73.2 (67.5-76.75)	72.4 (67.2-72.75)	72.5 (69.65-74.45)	–	–
Min – Max	65.4 – 83	62.3 – 78.1	64.2 – 86.5	–	–

## Discussion

This study evaluated and compared the clinical outcomes of three commonly practiced surgical techniques for pilonidal sinus disease, simple excision, Limberg flap, and keystone flap, in terms of operative parameters, postoperative morbidity, healing time, recurrence, scar quality, and QoL outcomes using the WHO QoL-BREF instrument. The mean age of participants was 25.2 years, which was consistent with previous reports by Karydakis (1992) [20] and Bascom and Bascom (2002) [21]. This might be explained by the occupational and lifestyle factors (e.g., prolonged sitting, obesity, poor hygiene) as reported by Tezel (2005) [22] and Al-Khamis et al. (2010) [11]. Marked male predominance (89.1%) was consistent with global studies reporting a male-to-female ratio of 3:1 to 5:1. This gender disparity is attributed to increased androgenic hair growth and deeper natal clefts in men. Most patients were students (50%) and had sedentary habits, which agrees with the mechanical theory described by Bascom (1980) [23] and Hodges' acquired theory [24], which holds that friction and hair insertion initiate the disease process [2,25]. The low prevalence of systemic disease reflects the cohort's younger age distribution and likely contributed to the generally favorable postoperative recovery.

Simple excision showed good results in limited disease (p < 0.001), and flap techniques for more extensive or recurrent cases, consistent with previous studies done by Mentes et al. (2004) [26] and Kepenekci et al. (2010) [27], as flap procedures reduce recurrence by flattening the natal cleft. Larger defect sizes in the Limberg and Keystone groups further support the use of flap procedures for wider excisions requiring tension-free closure, as shown by Bessa (2013) [28] and Guner and Cekic (2015) [29].

The Limberg flap had the longest operative time, followed by keystone and simple excision; similar findings were reported by Al-Khamis et al. (2010) [11] and Doll et al. (2007) [30], who attributed this to the greater dissection and inset required in flap procedures, though low recurrence and improved long-term outcomes justify the increased duration.

Hospital stay was significantly longer for flap procedures than for simple excision, consistent with findings by Akin et al. (2008) [13] and Karydakis (1992) [20], highlighting the trade-off between prolonged recovery and reduced long-term morbidity. This study shows a significant difference in wound healing time among the three procedures (p = 0.031). The faster recovery observed with flap techniques can be explained by their off-midline closure, which provides better blood flow, reduces wound tension, and promotes quicker tissue repair. In contrast, wounds following simple excision are left open to heal by secondary intention, leading to slower epithelialization and delayed healing. These similar findings have also been reported by Mentes et al. [26], Milone et al. [31], and Arumugam et al. [32]. Return to work was earlier after simple excision than after flap procedures, consistent with that reported by Pronk et al. (2019) [33], who noted faster mobilization with less extensive wounds but cautioned about a higher recurrence risk with midline approaches.

Postoperative complications occurred in 43.5% of patients, most commonly seroma (28.3%), with higher rates in Limberg and Keystone groups, while recurrence (13.3%) was seen only in the simple excision group (p < 0.001). This reflects the trade-off between early morbidity in flap procedures and later recurrence with simple excision, consistent with findings from Al-Khamis et al. (2010), Bascom and Bascom (2002), and Mentes et al. (2004), who also reported similar seroma rates in Limberg flap series [11,21,26]. In this study, recurrence (13.3%) occurred exclusively in the simple excision group, with no recurrences in either flap group (p < 0.001). This aligns with that by Karydakis (1992) [20] and Milone et al. (2018) [31], who reported lower recurrence rates with off-midline flap techniques, and Doll et al. (2007) [30], who emphasized the role of wound position (midline vs. off-midline) in influencing recurrence. Scar assessment using the Vancouver Scar Scale showed significantly better outcomes with flap procedures (p = 0.014), with Limberg and Keystone flaps producing flatter, more aesthetic scars and less discomfort on sitting, consistent with Guner and Cekic (2015) [29] and Bessa (2013) [28].

Patient satisfaction scores were highest in the Limberg flap group, in agreement with that reported by Al-Khamis et al. (2010) [11] and Müller et al. (2011) [34], who also reported greater satisfaction due to better cosmesis and lower recurrence rates. Although there was no significant difference in the distribution of Clavien-Dindo grades across the groups (p = 0.309), minor problems (Grades I-II) were more common, indicating that all three procedures are safe when used with proper postoperative care.

QoL assessment using WHOQOL-BREF demonstrated significantly better physical domain scores (pain, mobility, sleep, daily activities) and psychological domain scores (body image, self-esteem, satisfaction with healing) in the Limberg and keystone groups (p < 0.05), likely due to reduced recurrence, better mobility, reduced pain, off-midline closure, and improved scar outcomes. In contrast, social (personal relationships, social support) and environmental domains (financial resources, access to care, living conditions) showed no significant differences, suggesting limited influence of surgical technique on these broader aspects of QoL, as also reported by Milone et al. (2018) [31], Meinero et al. (2014) [25], and Pronk et al. (2019) [33], who concluded that flap reconstruction enhances QoL by minimizing pain, flattening the natal cleft, and preventing reinfection.

Thus, this indicates that although flap procedures involve longer operative and recovery periods, they offer superior long-term outcomes in terms of cosmetic appearance, wound durability, and overall patient satisfaction. Hence, the current study reinforces the global trend favoring off-midline flap reconstruction as the procedure for complex or recurrent pilonidal disease.

## Conclusions

This prospective comparative study demonstrates that while simple excision offers shorter operative time, earlier discharge, and faster return to work, it is associated with higher recurrence rates and less favorable scar outcomes. In contrast, Limberg and keystone flap reconstructions, though requiring longer operative time, hospital stay, and drainage duration, provide superior long-term results with lower recurrence rates, better scar quality, higher patient satisfaction, and improved physical and psychological QoL. Both flap techniques effectively flatten the natal cleft, reduce tension, and enhance cosmesis, reinforcing their role as the preferred options for complex or recurrent pilonidal sinus disease.

Thus, the findings support a tailored approach: simple excision for limited disease where rapid recovery is prioritized, and off‑midline flap procedures for extensive or recurrent cases, where durable healing, cosmesis, and QoL are paramount. This study adds to the growing evidence favoring flap reconstructions as the standard of care in complex pilonidal sinus management.
